# Looking beyond changes in averages in evaluating foundational learning: Some inequality measures

**DOI:** 10.1016/j.ijedudev.2021.102411

**Published:** 2021-07

**Authors:** Daniel Rodriguez-Segura, Cole Campton, Luis Crouch, Timothy S. Slade

**Affiliations:** a University of Virginia, United States; b Duke University, United States; c RTI International, United States

**Keywords:** Learning inequality, Foundational literacy, Learning poverty at bottom of pyramid, Learning measurement, Distribution of impact, Learning outcomes

## Abstract

This paper uses measurements of learning inequality to explore whether learning interventions that are aimed at improving means also reduce inequality, and if so, under what conditions. There is abundant evidence that learning levels are generally low in low- and middle-income countries (LMIC), but there is less knowledge about how learning achievement is *distributed* within these contexts, and especially about how these distributions change as mean levels increase. We use child-level data on foundational literacy outcomes to quantitatively explore whether and how learning inequality using metrics borrowed from the economics and inequality literature can help us understand the impact of learning interventions. The paper deepens recent work in several ways. First, it extends the analysis to six LMIC, displaying which measures are computable and coherent across contexts and baseline levels. This extension can add valuable information to program evaluation, without being redundant with other metrics. Second, we show the large extent to which the disaggregation of inequality of foundational skills between- and within-schools and grades varies by context and language. Third, we present initial empirical evidence that, at least in the contexts of analysis of foundational interventions, improving average performance can reduce inequality as well, across all levels of socioeconomic status (SES). The data show that at baseline, the groups with the highest internal inequality tend to be the groups with lowest SES and lowest reading scores, as inequality among the poor themselves is higher than among their wealthier counterparts. Regardless of which SES groups benefit more in terms of a change in mean levels of reading, there is still a considerable reduction in inequality by baseline achievement as means increase. These results have policy implications in terms of targeting of interventions: much can be achieved in terms of simultaneously improving averages and increasing equality. This seems particularly true when the initial learning levels are as low as they currently are the developing world.

## Highlights

• Reducing learning inequality in foundational literacy is possible.
• Interventions focusing on foundational skills may reduce learning inequality even without specific inequality targeting.
• Measurement of learning inequality in foundational literacy is possible using existing tools familiar to economics.
• Measuring learning at the bottom of the pyramid can lead to insights otherwise not observable through sample averages.
• Improvements in means and in learning equity are not only compatible but indeed tend to happen simultaneously.

## 1 Motivation

The learning crisis—estimated to affect about half of the children in low- and middle-income countries—is a well-documented phenomenon (World Bank, 2017; UNESCO Institute for Statistics, 2017). In spite of the impressive recent gains in enrollment, many pupils around the world spend a large portion of their childhood and teenage years in school without substantially improving their foundational literacy and numeracy skills. As a result, large swaths of children exit the educational system being functionally illiterate and innumerate. As such, the learning crisis is a policy issue deserving attention from policymakers and citizens. From the governmental perspective, large amounts of public funds are invested into educational systems with relatively little learning to show in return. From the household perspective, the opportunity cost of schooling is high for parents and children, especially those in extreme poverty. Increasing the return on all this sacrifice through the delivery of higher cognitive and noncognitive skills, which in turn can enable higher standards of living in the future ought to be a core function of education systems. This is especially true in low- and middle-income countries (LMIC) today where, unlike during the colonial past, mere credentialing, or actual education but only of a minority, is unlikely to ensure jobs or higher earnings. These severe educational deficits enforce and perpetuate intra-country inequalities in terms of educational achievement and mobility. In order to address these cognitive gaps and inequalities, informed research and policy requires deeper knowledge of the *distribution* of foundational skills within countries and sub-populations, beyond the more aggregated reports which support the existence of a learning crisis or that show that interventions can improve mean learning levels. In this study, we leverage detailed, individual-level foundational literacy data across six LMIC to characterize the distribution of foundational skills in detail, to show how that distribution is affected by progression in the school system and by factors such as socioeconomic status, and by certain programs aimed only at improving means, and to display what has happened to inequality when mean performance has increased.

This paper responds explicitly to three strands of current literature. Chronologically, the first strand of the literature is work on “learning at the bottom of the pyramid”, mostly associated with Wagner et al. (2018). The second line of research is the literature on inequality of learning, flat learning profiles and learning at the right level associated with Pritchett and others at the RISE Programme (see Pritchett and Beatty, 2015; Kaffenberger and Pritchett, 2020; Crouch et al., 2020). The third body of knowledge is the literature mostly associated with the World Bank around “learning poverty” (World Bank, 2019). A discussion of these literatures follows, interweaving the various strands of it. We foreshadow one important distinction between these three strands, subtly following what we say in the paper. The term “bottom of the pyramid” is usually associated with a concept of material deprivation or marginalization, and refers to populations who are not normally well reached by large, formal, “modern,” standardized systems (be it private sector corporations or education). The implied targeting criterion is by income or other vulnerability factors. The other two literatures, especially the learning poverty strand, refer to inequality of learning outcomes and emphasize cut points in learning as a way of targeting. Obviously, cut points in learning will tend to correlate with cut points in the income distribution or socioeconomic status (SES), but as this paper will show in detail, they are definitely not the same construct. The RISE literature tries to bring the various strands together, more recently with an emphasis on pedagogical coherence. This paper tries to work with all these distinctions but, given the provenance of the paper, it tries to work mostly with the strand on pedagogical coherence. Our use of terms such as “bottom of the pyramid,” “learning poverty,” and others is meant to link to these literatures. It should be noted that these literatures are themselves not always cognizant of how, precisely, they talk to each other.

Beyond the overall low learning outcomes in LMIC, a volume of recent research has documented the existence of persistent inequalities and inequities in educational achievement (Crouch et al., 2020). Glewwe et al. (2009) describe the strong elite-bias of the Kenyan educational system inherited from colonial educational structures, and the skewed incentives that teachers have to cater their education towards the top achieving students. Similarly, Muralidharan et al. (2019) describe how the entry of a large number of first-generation primary school students into the Indian educational system as a result of the increase in overall enrollment has also boosted within-school and within-class inequalities, with students from a more diverse background now going to school. These first-generation students are more likely to drop out of school, and perform worse in school (Portela and Atherton, 2020), which ultimately increases within-class inequality. On top of this, there is work, such as that of Pritchett and Beatty (2015), which describes the deep mismatch between fast-moving and wide-spanning curricula, and the baseline achievement of children. In contexts where pupil-teacher ratios are very large, catering to the full range of achievement becomes an extremely challenging task (Duflo et al., 2011), effectively forcing teachers to choose who to teach. The combination of “overambitious curricula”, large classes with heterogenous age and skill distributions, instruction in colonial languages, and structural incentives that reward “teaching to the top” can yield low and unequal learning levels. All these factors together can result in already high-achieving students benefiting even more from schooling, possibly exacerbating existing inequalities at the time of entry into school. This type of detailed analysis of inequality and its progression throughout the different grades needs to be more carefully developed, as we do in the current paper.

In light of the learning crisis, researchers and policymakers have started to shift their focus from increasing enrollment and years of schooling to raising learning for all children. Furthermore, some of these efforts have also pivoted towards measuring equity and equality, beyond just tracking average performance and access within specific geographic regions, as required by Sustainable Development Goal 4, with its distributional emphasis (United Nations and Economic and Social Council. Statistical Commission, 2016).^1^ Important work has developed a better understanding of the types of educational policies that enable higher overall learning, as we review below. Most of this work has focused on identifying the local constraints for learning, and relieving these barriers through scalable approaches. For instance, one strand of work has focused on better matching between class instruction and students’ starting level and pace of learning, such as the Teach at the Right Level interventions in India and Sub-Saharan Africa (for instance, Banerjee et al., 2017). By tailoring instruction to what students already know, and how quickly each student moves to higher achievement levels, schools can ensure that students are never too far behind or ahead. Furthermore, this type of tailored instruction can reduce classroom inequality through approaches that group students by achievement rather than by age, enabling teachers to better cater to more homogenous classes. Approaches in the same spirit such as Room to Read in India (Joddar and Cooper, 2017; Joddar, 2018), or PRIMR and later on Tusome in Kenya (Piper et al., 2015, 2017, 2018a, 2018b), have also provided evidence that by relieving the constraint of poorly targeted instruction, children’s learning can improve significantly.

Other comprehensive interventions, such as Eble et al. (2019) in Gambia, implement improvements to instruction delivery via teacher coaching, tutoring, and teacher scripts. The promising findings of this study support the hypothesis that improvements in instruction quality can significantly increase learning levels. Furthermore, improving instruction can take many forms and must be tailored to the local context. Work in Pakistan by Beg et al. (2019) shows that videos with expertly-delivered content that fill in teachers’ knowledge can be an effective and scalable approach to improving instruction. Finally, there have been many attempts to relieve constraints via traditional input interventions (e.g., more books, more teacher training), or more traditional “governance” interventions such as school-based management or more parental involvement, but, on average, these “governance” interventions have not been as effective at raising learning outcomes, compared to those that target instructional and pedagogical approaches. (Crouch and DeStefano, 2017; Evans and Yuan, 2019). Even in terms of reducing inequalities, Zuze and Leibbrandt (2011) find that under the right conditions, policies that promote physical resource availability could amount to equity gains but also that, in general, equalizing access to education does not guarantee more equitable outcomes. Even within this category of interventions, appropriately designed policies can still be plausible avenues. For instance, work in Tanzania by Mbiti et al. (2019) shows that combining teaching incentives with school capitation grants to ensure that teachers are motivated and have the physical inputs to deliver quality instruction, can improve learning outcomes. The current body of impact evaluation research briefly reviewed above has been extremely valuable, as these new solutions start informing broader policy plans, such as the large-scale implementation of Teach at the Right Level in Botswana (TaRL, 2020).

Unfortunately, a blind spot in this general body of work is the little attention paid to thoroughly characterizing the changes in the full underlying distribution of foundational skills induced by these policies (Gruijters and Behrman, 2020). Research studying the previous implementations of these programs which mostly targeted, and were successful at, improving the *average* results, did not necessarily focus on descriptions of the initial distributions or their shifts post intervention. To be fair to the current literature, there is substantial focus on analyses by sub-groups of policy interest through specifications that test for heterogeneous effects by baseline performance, gender, region, grade, among others. However, the main focus of these analyses was some version of aggregated treatment effects by these sub-groups, rather than on analyses of the complete distributions.

Some work has started to explore cross-sectional changes in inequality, but more thorough and longitudinal studies of inequality still need to be better developed. As an exception to the lack of longitudinal analyses, a paper by Crouch and Rolleston (2017) puts forth evidence from regional learning assessments and special longitudinal studies that measure learning in the same group of children as they grow older (studied through SACMEQ and Young Lives). Similarly, Crouch et al. (2006 uses household surveys from most of Latin America to look at intergenerational changes in inequality, finding that the Gini coefficient for years of education improved significantly from 0.58 to 0.36. Other studies (Crouch and Rolleston, 2017;Crouch et al., 2006) provide some initial evidence that countries that go from very poor mean performance to middle mean performance do so by reducing the percent of students at very low levels. In this sense, work such as Gruijters and Behrman (2020), and Oketch et al. (2020) has pointed out that raising overall outcomes, and decreasing dispersion is desirable, as long as this shift comes from “raising the floor”, and not at the expense of higher performers. Even earlier work by Willms (1999) identified socioeconomic gradients in literacy, where countries with the highest literacy rates also having the flattest gradients. Taken together, these papers highlight that relative literacy equality tends to benefit students across the full distribution. In fact, these studies provide initial evidence that countries that experience increases in learning from low average levels to middle average levels, do so by shifting the distribution from the left to the middle, without significantly affecting the right tail of the distribution. Beyond these studies, not much research explores long-term changes in the inequality coefficients. Therefore, in spite of how entrenched and critical intra-country educational inequality may be for educational systems and labor markets, researchers have not yet explored a set of harmonized tools to characterize the underlying skill distributions.

There have been some calls to pay attention to educational inequality, particularly at the “bottom of the pyramid”, from scholars like Dan Wagner et al. (2018), by proposing a Gini coefficient for education, in the context of the more foundational skills. Unfortunately, this work has not yet reached the mainstream of impact evaluation education research in LMIC. Looking at broader descriptive work, international assessments like ASER or Uwezo have been instrumental at providing evidence for the magnitude of the learning crisis. In fact, PAL and EGRA^2^ sorts of measures have now been used in hundreds of country/language/script contexts.^3^ The rapid and basic nature of these assessments has been pivotal to scale them at the level of countries. However, it is important to note that these assessments focus on emerging or foundational literacy, and not as much on the deeper comprehension tasks that are more commonly measured towards the end of primary schooling.

Understanding the intra-country distribution of skills in depth is also critical for policy design. Countries aiming to raise standards must ensure that the median or modal individual student gains knowledge, in addition to students collectively gaining knowledge on average. The distinction here is that improvement in a small portion of exceptional students may drive an average improvement, even if the median or modal student does not see any improvement. It is not a mathematical necessity that improving averages would reduce inequality. Thus, if efforts that focus solely or mostly on improving means can also drive reductions of inequality, that is a meaningful or non-trivial result. After all, raising outcomes for students at the low end of the distribution will result in the same overall average gain as raising outcomes by the same amount for a similarly sized group at the top of the distribution. Therefore, understanding the relative sizes of these portions of the distribution, and how they change with interventions that are successful at raising the mean, will illuminate the extent to which differentially targeted policies can raise average country-wide outcomes. Furthermore, understanding the distribution of skills not only by baseline skills purely, but also by other important characteristics like socioeconomic status, gender, and geographic clusters allows for a better-informed set of policies. In particular, understanding how inequality manifests in the broader population can inform whether average-improving policies should be targeted at specific groups, schools and regions, or whether broader reform is needed to have a wider reach. Finally, there is evidence that not all gaps in achievement are a result of differential school experiences, but also that home and community environments can play an important role in shaping school performance (Chetty and Hendren, 2018a, b; Doyle et al., 2017; Heckman and Karapakula, 2019). Given the recent influx of first-generation students, the distribution of achievement at the time of entry into the formal educational systems has likely widened. By understanding how learning is initially distributed and how it evolves during the first years of schooling, policymakers can design effective policies that fill in the resource gaps which end up compounding achievement gaps for different groups of students.

The paper proceeds as follows: section II introduces the broader concept of inequality and the methodological approaches to measure it; sections III-V describe certain types of quantitative analyses across six countries which can be performed to characterize inequality; section VI provides some limitations to our methods and contributions; and section VII concludes by exploring some of the pedagogical underlying causes to such inequality mostly as suggestions for further research.

## 2 An understanding of inequality for the purposes of this paper

As previously noted, the large volume of research on interventions that improved *average* educational outcomes has not focused as much on the distributional changes that may have happened during the course of each intervention. Similarly, little is currently known about how foundational skills are distributed throughout the most disadvantaged populations in LMIC. Understanding inequality in learning outcomes is key to designing policies which boost overall learning while also improving equity. Given these relatively large gaps in the foundational learning literature within LMIC, we will consider different angles from which inequality can be described. These different approaches, which include various measures of inequality and dispersion, will allow us to be more precise about where outcomes are most unequal, and how these might evolve over time as average skills improve.

### 2.1 Definition

There is still a need for the literature to consider more rigorously the distributional changes of interventions. In order to understand these distributional changes, and resulting inequality, we must be clear about the working definition of “inequality.” From a positivist point of view, we will define inequality as the extent to which children with a common characteristic (e.g., grade, school, or even country of origin) perform differently in a given task, which in our case will be foundational literacy, as measured by oral reading fluency. Inherently, any measure of inequality quantifies dispersion of the overall outcome data, as opposed to the computation of statistics like the median or the mean. Instead, inequality is concerned with how *far apart* similar children’s learning levels are, especially relative to the mean level. Within this definition, there are several substantive mathematical tools to quantify the actual distance among children.

From a normative point of view, much policy and philosophical discussion has been geared towards addressing whether inequality per se is a worrying outcome, especially in terms of material well-being (e.g. income or wealth). In this sense, inequality must not be confused with inequity. Inequity relates to the concept of social justice, and carries negative implications of unfairness. Instead, inequality may or may not be a product of inequity, but it may also arise due to other circumstances such as random chance (e.g. testing issues), weak application of quality standards and quality assurance, or innate differences in ability. While part of the inequalities that we describe do indeed come from inequities in human capital development along lines of gender (Jayachandran and Pande, 2017), ethnicity and regionality (Ejdemyr et al., 2017), or language of instruction (Glewwe et al., 2009), among others, disentangling what portion of a given measure of inequality comes from which inequities is a challenging and messy task, which we will not attempt. Still, some of these may be more obvious than others: for example, gender inequality is a more recognizable and quantifiable form of inequity than inequality based on poor standards (e.g., the bad luck of having a “bad” teacher in a system that does not effectively guarantee a minimum to teacher—or teach*ing*—quality).

**Fig. 1:**
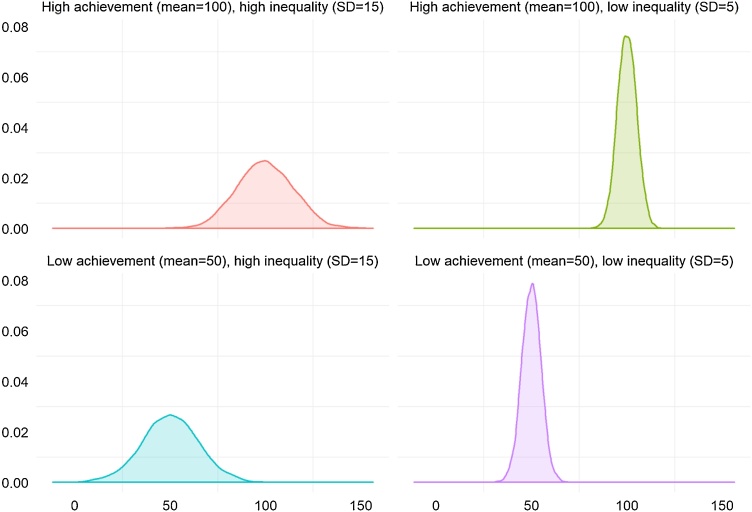
Simulated distributions with variations of high/low achievement and high/low dispersion. **Notes:** the data shown is the result of four different simulations with n=5,000 each, for illustrative purposes only. Each simulation comes from a random number generator drawing from a normal distribution, with the mean and standard deviation specified above each panel.

Our only prescriptive argument regarding inequality is that, for educational purposes in the earlier grades, ceteris paribus, less inequality is better than more inequality. Fig. 1 illustrates this point by also layering an additional lens of mean achievement onto inequality, as measured by different degrees of dispersion. The argument we make for the top row is that conditional on having the same mean, a narrower distribution is preferable as it allows teachers and schools to target instruction and resources for the more specific level where children are.

This is consistent with the literature on teaching at the right level and “over-ambitious curricula” noted above. The normative case for which of the two cases on the bottom row is preferable is tougher, as there could be advantages for a country of having high performers even if the overall mean is low. However, from the point of view of intervening to raise outcomes, the case with low means and low dispersion also provides an opportunity to target a level that will reach many children. In the case of low mean and high inequality, as displayed by the bottom left quadrant, policymakers might have to make an explicit or implicit decision to target the top or bottom portions of the distribution, unless it is the case that certain types of programs are in some sense self-targeting to the left hand side, an issue that we hope to better inform through some of our findings in this paper.

### 2.2 Types of inequality

For analytical purposes, we also need to distinguish between two key types of inequality: “pure” inequality and inequality by other characteristics. Pure inequality refers to the inequality that stems from different achievement levels. It is typically quantified by identifying features of the underlying distribution of achievement, such as measures of dispersion or the comparison of arbitrary points on the distribution. Pure inequality is assumed to be at least partly generated through randomly allocated factors such as innate skills at the time of birth or indifferent teaching (where some children might be in luck and get a fairly good teacher, and others are out of luck). Mathematically speaking, it represents the largest possible inequality, as any other sub-group analysis is by definition a sub-sample of the broad distribution described by pure inequality. Pure inequality is also, from a pedagogical point of view, the micro-level reality of many teachers on the ground that need to cater to students within a class with its own level of pure inequality.

The second type of inequality refers to inequality by individual or community-level characteristics, such as socioeconomic status, gender, language, ethnicity, or province. This type of inequality is usually reported through sub-group analyses, and comparisons of summary statistics across sub-populations of interests. Furthermore, this type of inequality often deeply molds the distribution described by pure inequality measures. This type of inequality has gotten more attention in the literature for at least two key reasons. First, it may be more actionable than pure inequality. For example, identifying the students for large-scale interventions intended to narrow gaps due to, for instance, ethnic groups may be more actionable and pragmatic than identifying interventions aimed at the bottom 20 % of students nationally. Secondly, this type of inequality is much more often associated with connotations of inequity than pure inequality. Still, policy-makers should have strong theoretical and practical reasons to pay attention to both types of inequality.

### 2.3 Measures of inequality

In an effort to quantitatively describe inequality, there are several descriptive metrics available to researchers for this purpose. For the most part, these measures are borrowed from the economics literature interested in inequality and variability. The specific metrics we explore are the Gini coefficient, the coefficient of variation (CV), different P_x_ to P_y_ ratios, and the percent of children scoring at zero.^4^ Table 1 presents further details of what these measures are, and their respective strengths and weaknesses. Furthermore, it is important to clarify that this is by no means a comprehensive list of measures of inequality. We purposefully curated a list of measures that could both reliably inform about the shape and position of the underlying distribution, but that could also be intuitively understood by thoughtful policymakers and researchers in fields that do not use these measures as often. For example, the “generalized index of entropy with α=x” has desirable theoretical properties, such as more control over which portions of the distribution have a heavier weight on the measure of inequality at the time of producing one summary figure. However, it is not a well-known measure, nor does it have an intuitive “natural connection” to the data in the way that other metrics such as P_x_ to P_y_ ratios might. Therefore, we decided to not include measures such as this one.

**Table 1:** Inequality measures explored in this paper.

Measure	Description	Strengths	Weaknesses
Gini coefficient	A measure of relative inequality expressed as a coefficient ranging from 0 to 1. A value of 0 represents a situation in which all individuals own an equal amount of the good in question (in this case, “learning” as represented by ORF). A value of 1 represents a situation where one individual owns all of the good in question (and no others own any). If all ORF scores increase by the same absolute amount, the Gini coefficient decreases even if the absolute distance between the highest- and lowest-scoring individuals is the same as before, as it is computed relative to the overall level.	The measure is well-known to both economists and non-economists, and has been the most used measure in education thus far (albeit generally applied to years of schooling rather than reading outcomes). Any Gini coefficient has a graphical equivalent in the Lorenz curve, and the Lorenz curve, in turn, can be used to visualize where in the distribution the inequality comes from.	No “natural” translation to the magnitude of the measure. Particularly sensitive to high outliers, particularly when the overall mean is low.
Coefficient of variation (CV)	Standard deviation over the mean. It is bounded below at zero and has no theoretical upper bound. As with the Gini coefficient, an equal absolute increase in ORF scores for all individuals would drive the measure lower.	Easy to calculate. No specialized substantive or computational knowledge required. It has a “natural” interpretation in the data.	Breaks down when the mean is zero (for instance, with normalized data). Being a ratio of two numbers, a change in CV does not immediately reveal which of the two (or both) moved. No well-established general bounds.
Ratio of P_x_ to P_y_, typically 90^th^ to 10^th^ or 75^th^ to 25^th^.	Ratio of the score recorded by the person(s) at the x^th^ percentile of the outcome distribution to the score recorded by the person(s) at the y^th^ percentile of the same distribution. It has a lower bound of 1, no upper bound, and is a relative measure in the same way as the others.	Intuitively appealing and commonly used. Analogous to the popular economic and political literature around “% of wealth possessed by the 1 percent-ers.”	Breaks down when the lower P_y_ is 0. Even if ratio is flipped to avoid dividing by zero, uninformative as the result would always be zero regardless of denominator. No well-established general bounds.
Percent scoring zero	This is not strictly a measure of inequality; rather, it is the proportion of students unable to read a single word. It is a stark, visceral indicator of poor learning outcomes that is both intuitive and effective in catalyzing institutional effort to remedy.	Very easy to interpret. Commonly used in EGRA reports as a metric that can be appropriately compared across languages and scripts without encountering the dangers of comparing ORF scores across scripts or language families.	Only provides a snapshot of two portions of the distribution, hence does not characterize the full distribution.

**Notes:** table adapted from Crouch and Slade (2020).

The importance of at least initially exploring the issue of inequality through more than one “preferred” metric arises from two fronts. Firstly, no single measure can be expected to reliably describe all features of a distribution which could be of interest to researchers and policymakers. In this sense, none of these measures is a perfect reflection of learning inequality. Hence, analyzing inequality through diverse lenses, each with their own strengths and weaknesses, is likely to provide a more well-rounded understanding of skills at the bottom of the pyramid (in an income sense) or among the learning poor (in terms of learning thresholds). Secondly, to the best of our knowledge, these measures have not been fully tested on the type of data we use to describe learning at the bottom of the pyramid. Hence, exploring more than one measure across different settings and datasets can provide a better sense of the empirical characteristics of each of these metrics on this type of data.

### 2.4 Instrument and data used, context for the data-gathering

The inequality analyses presented in this paper are based upon the oral reading fluency (ORF) subtask of the early grade reading assessment (EGRA) battery of measures. The EGRA is a one-on-one, face-to-face, live oral reading assessment, conducted by an assessor having a child read in response to a set of stimuli (familiar words, a connected text passage, letters to be sounded out, etc.) and then recording the child’s responses on a paper or electronic response form.^5^ The assessment focuses on skills that are foundational, such as knowledge of the alphabet and letter sounds, ability to read familiar words, non-familiar or invented words that follow the orthographic rules of the language in question (to assess decoding rather than memorization), ability to read a connected text passage, and answer a few comprehension questions. The assessment takes about 20 minutes. It is in the public domain and therefore not all applications are well documented. Still, more or less “official” applications with known quality control features include some 150 country/year/language combinations, usually in two or three grades per case (most often grades 2 and 3, but sometimes including grades 1, 4, and 6), in dozens of languages (the assessment is applied in the language of instruction in the foundation grades) and many non-Latin scripts. For the purposes of this paper, we leverage EGRA data from six different countries. These six countries were chosen because the datasets from the countries had data on SES, had both baseline and end-line measures from an intervention, and had many languages, or some important combination of those factors. Table 2 provides a description of the data sets used.

**Table 2:** Description of data used for this study.

Country	Number of students (schools)	Type of data (panel/ repeated cross-section/ snapshot)	Grades	Language(s)
Democratic Republic of Congo	2346 (230); 7081 (290)	Unique round; Baseline/Endline	3; 4, 6	Lingala, Tshiluba, Kiswahili; French
Egypt	2118 (56)	Baseline/Endline	2	Arabic
Kenya	12,769 (302)	Baseline/Midline/Endline	1, 2	English, Kiswahili
Malawi	5120 (173)	Baseline/Unique rounds	1, 2, 3	Chichewa
Philippines	6414 (308)	Baseline/Endline	1,2	Cebuano, Ilokano, Hiligaynon, Maguindanaoan
Uganda	12,146 (620)	Baseline/Endline	1−6	English, and 12 local languages like Luganda, Acoli, and Lugwere

**Notes:** the data from the Democratic Republic of Congo comes from two different projects. The details for each project are separated by a semi-colon.

One advantage of the EGRA is that, similar to those assessments in the PAL network such as ASER and UWEZO (and PISA for that matter), the same assessment is used in all grades, so one can observe the flatness of the grade-wise learning profile, and therefore differential performance by grade is attributable to something about the learning levels and processes, not a difference in the assessment. The pedagogical and policy uses of EGRA are explained in the literature (see, e.g., Dubeck and Gove., 2015; Gove et al., 2015). A toolkit that explains sampling approaches and application procedures is available (RTI International 2016). Gove et al. (2017) describe how the assessment has been used in impact evaluation of reading programs similar to those used for this paper, and the nature of such programs. Several psychometric descriptions exist—a fairly typical and thorough one (using Spanish as a case in point but generalizing thoroughly) is Jiménez et al. (2014).^6^ As a way of characterizing the sorts of interventions and their usage of EGRA or similar assessments, Graham and Kelly (2017) summarize more than two dozen such interventions and document a median effect size on ORF of 0.45, equivalent to boosting learning (in these terms) by about 2 grade-equivalents. Thus, these are significantly impactful interventions in terms of mean effects—useful background that prompts to ask whether they might also have an impact on equality. Piper et al. (2018a, 2018b) characterize successful interventions as typically consisting of structured lesson plans, teacher coaching, better, plentiful, and inexpensive books, appropriate assessment (such as, for these purposes, EGRA or something like it), time on task, all with a focus on reading per se, not “language” or language arts. Crouch and DeStefano (2015) note how these various inputs have to be very tightly and intensely managed and coordinated, and how such management needs to be based on reading outcomes data.

### 2.5 Outcome of interest

Among all the sub-skills measured by the EGRA, we chose “oral reading fluency” (ORF) as the main outcome of interest. ORF is the closest analogue in the current EGRA battery to the key skill of independent reading of narrative text, and to the more colloquial meaning of “being literate.” Similarly, ORF is usually the skill that is most correlated with the other skills in the assessment, and has the highest loading on a principal component analysis (Jiménez et al., 2014) of the assessment. ORF scores are quantified in correct words per minute (cwpm) and are calculated by tallying the number of words the student has correctly read aloud and dividing that sum by the proportion of time elapsed in seconds. Specifically, correct words per minute is measured as follows:

$$\text{cwpm}=\frac{\text{words correct}}{\text{(time elapsed}\text{ in seconds}\text{)}\text{/}60}$$

In particular, ORF exhibits several useful characteristics which make it well-suited for these analyses. First and most importantly, it is a continuous measure with a large range, typically characterized by an absolute minimum of 0 and no theoretical maximum.^7^ Second, it is easily interpretable: an ORF of 60 cwpm represents a child reading one word per second, a cadence which is easy for audiences to model and evocative to listeners. Third, it is empirically meaningful: fluency is highly correlated with reading comprehension, which is the most salient reading skill for broader educational achievement.^8^ Note that this paper does not assert that reading and learning are synonymous.

We also do not claim that oral reading fluency is the best measure of whether a child is truly reading, nor that ORF is the best measure to estimate the metrics that we explore. We select ORF as a reasonable proxy for reading skill because it is well correlated with comprehension, relatively straightforward to measure with fidelity (as compared to, say, silent reading), and available for a wide range of languages in a wide variety of contexts since reading-skill assessments in low- and middle-income countries began to grow in popularity around 2010 (RTI International, 2015).^9^ ORF as a metric also poses certain methodological challenges. Unlike alphabetical knowledge or phonological awareness, ORF is beyond the earliest of “emergent” literacy skills and as such, depending on the grade-targeted and context, a more advanced measure like ORF could yield “floor effects” by placing many children at 0. These floor effects would hinder the researcher’s ability to distinguish amongst these students (even if, for instance, the number of letters they can recognize is indeed different). This clearly has implications for the “percent scoring zero” metric as well: even within the same sample, more advanced literacy tasks like ORF will yield higher shares of children at zero than emergent literacy skills like alphabetic knowledge. We also acknowledge that had we chosen another more emergent skill, the opposite could have happened by reaching ceiling effects in certain sub-populations (in an extreme example, measuring letter names among 12th graders). Ultimately, we do not advocate for the universal use of ORF as the key measure to compute inequality metrics, as this is a highly context-dependent decision. Our use of ORF partly reflects a need to choose a common metric to showcase our analysis across all samples used in this paper. Similarly, the potential presence of floor or ceiling effects in any of our samples is not necessarily a weakness of the metric in question, but rather of the choice of skills used to estimate the inequality metrics.

Note that an issue with measuring inequality with this type of achievement data is that they do not necessarily follow as simple and clear-cut distribution as other educational outcome data do, by circumstance or by design, such as, PISA or TIMSS. Therefore, the simple characterization of the distribution of early literacy or numeracy skills through the computation of a mean and a variance may not be enough to understand what the distribution looks like, as it would in more regular distributions. There is a clear need to explore and utilize other (and varied) methodological tools and metrics to understand inequality in these contexts, as we do in this paper.

## 3 The performance of different measures of inequality

Using data on oral reading fluency across six countries, we empirically test the different metrics described in Table 1, as shown in Table 3. Specifically, Table 3 is meant as an illustration of how these indicators perform, and not as a comprehensive display of all possible sub-populations in these data sets.^10^ Instead, this table should serve as a pattern-seeing tool to evaluate the appropriateness of these metrics to measure what students in these samples and contexts know, and how this knowledge is distributed. Specifically, through this table we would like to understand how these metrics “behave”, i.e. whether they yield interesting and meaningful values when applied to these data. Below we go through a description of how each metric behaves, and how they behave together—do they cohere to give a fuller picture?

**Table 3:** Computation of inequality measures for select subsamples across six LMIC.

Country	Language	Grade	Phase	Mean	Gini	CV	p90/p10	p75/p25	% zero
Democratic Republic of Congo	French	4	Baseline	8.1	0.755	1.7	.	.	59.4
			Endline	9.0	0.749	1.7	.	.	54.4
		6	Baseline	27.1	0.467	0.8	.	12.8	23.3
			Endline	32.5	0.458	0.8	.	5.7	16.4
	Kiswahili	3	Unique round	1.6	0.895	2.9	.	.	80.5
	Lingala			1.8	0.899	3.0	.	.	78.7
	Tshiluba			2.5	0.875	2.6	.	.	77.2
Egypt	Arabic	2	Baseline	10.3	0.701	1.6	.	.	47.6
			Endline	18.7	0.661	1.4	.	.	34.2
Kenya PRIMR	English	1	Baseline	6.5	0.799	2.0	.	.	62.4
			Midline	26.4	0.543	1.0	.	21.5	23.9
			Endline	29.1	0.522	1.0	.	16.7	21.6
		2	Baseline	25.1	0.546	1.0	.	.	25.8
			Midline	46.1	0.411	0.7	.	3.3	10.1
			Endline	54.5	0.363	0.6	25.3	2.7	8.5
	Kiswahili	1	Baseline	4.4	0.809	2.0	.	.	68.9
			Midline	18.6	0.482	0.9	.	9.7	21.9
			Endline	18.9	0.499	0.9	.	.	25.8
		2	Baseline	17.8	0.522	0.9	.	.	30.7
			Midline	30.1	0.359	0.6	.	2.4	10.4
			Endline	32.6	0.338	0.6	14.3	2.3	8.9
Malawi	Chichewa	1	Baseline	0.2	0.980	6.7	.	.	97.3
		2	Unique round	1.0	0.948	4.2	.	.	91.0
		3		2.7	0.900	2.8	.	.	83.8
Philippines	Cebuano	1	Baseline	21.1	0.535	1.0	.	17.5	22.2
			Endline	21.9	0.495	0.9	.	35.0	23.4
		2	Baseline	39.8	0.338	0.6	23.6	2.5	7.8
			Endline	44.7	0.285	0.5	9.1	1.9	4.6
	Ilokano	1	Baseline	14.7	0.574	1.1	.	.	33.3
			Endline	17.7	0.497	0.9	.	9.7	17.4
		2	Baseline	30.1	0.375	0.7	.	3.7	12.9
			Endline	33.4	0.329	0.6	28.7	2.3	9.9
	Hiligaynon	1	Baseline	12.9	0.660	1.3	.	.	44.2
			Endline	13.7	0.639	1.2	.	.	35.6
		2	Baseline	31.6	0.451	0.8	.	12.5	21.7
			Endline	27.0	0.519	0.9	.	45.0	24.6
	Maguindanaoan	1	Baseline	6.9	0.782	1.8	.	.	66.3
			Endline	8.2	0.751	1.6	.	.	60.4
		2	Baseline	20.9	0.547	1.0	.	.	38.0
			Endline	22.8	0.485	0.9	.	.	28.6
Uganda	English	1	Baseline	0.2	0.984	8.3	.	.	96.3
			Endline	0.7	0.950	4.5	.	.	89.1
		2	Baseline	3.0	0.874	2.8	.	.	72.8
			Endline	5.7	0.835	2.2	.	.	67.3
	Luganda	1	Baseline	0.2	0.988	9.0	.	.	97.7
			Endline	2.1	0.905	2.9	.	.	86.3
		2	Baseline	6.2	0.788	1.8	.	.	65.4
			Endline	10.4	0.672	1.3	.	.	48.5
	Acoli	1	Baseline	0.0	0.994	17.0	.	.	99.2
			Endline	0.3	0.969	5.6	.	.	95.8
		2	Baseline	0.5	0.975	6.2	.	.	93.8
			Endline	3.3	0.907	3.0	.	.	84.1

**Notes:** “Mean”: average number of correct words per minute. "CV”: coefficient of variation; “% zero”: Percent of children at 0 correct words per minute. A dot in a cell means “undefined.”.

### 3.1 The performance of different metrics

The first interesting feature of these data is the wide diversity, especially across samples, in terms of oral reading fluency (ORF). English scores are close to zero for the earlier grades in the Uganda and Malawi samples, but they are closer to 40 words per minute for Cebuano in the Philippines sample. Therefore, it is important to keep in mind that given that the outcome variable (correct words per minute) is bounded on the left by zero, the shape of each distribution will be invariably affected by the proximity of its mean to zero. Having said this, the direction in which the relative position of the mean will influence inequality is ambiguous.^11^ It could be the case that means closer to zero represent a distribution where everyone is equally low, or, instead, it could be that averages which are further from zero allow more individuals to have at least some of the “good” (oral reading fluency) in question, decreasing inequality. We therefore also aim to understand the relationship between mean achievement and inequality for this type of foundational literacy outcome.

**Fig. 2:**
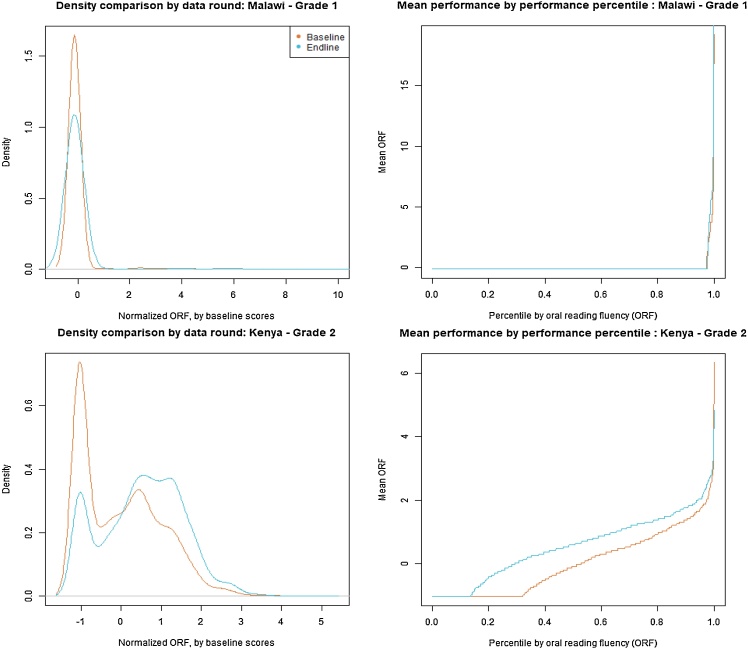
Distributional comparisons of oral reading fluency across countries. **Notes:** the left column displays the baseline and endline distributions, normalized using the baseline data means and standard deviations. The right column displays the mean oral reading fluency (as measures by correct words per minute) by percentile.

Note that the mean, here, is not a measure of inequality and is in the table only to indicate where the distribution is “centered,” an important issue to consider in interpreting the inequality measures. Aside from these summary measures in Table 3, one could also utilize tools that do not require the collapse of different distributional features into a single feature. For example, Fig. 2 shows a visual analysis which describes certain properties of the kernel distribution while maintaining interpretability, for two different contexts. Specifically, the column with panels on the left simply shows the distribution of normalized scores at baseline and at endline (using the baseline parameters) for two studies. Therefore, if a given intervention had positive and equal distributional effects across the full sample, the endline density would appear shifted to the right, relative to the baseline density. In practice, changes do not need to happen equally across the full distribution (i.e., through perfect rightward shifts), as the Kenya panel (bottom left) shows. Instead, these changes are driven by a large improvement across a large section of the middle distribution. This approach also allows to see whether no substantial distributional (or even average) effects happen, such as in the case of Malawi. These plots can then be translated into cumulative distributions that show the underlying values at each percentile. Interestingly, the two examples we show display very different characteristics: in Malawi, over 90 % of all children achieve 0 cwpm, and there is no change across testing rounds—clearly not a very successful intervention on just about any score. In Kenya, instead, a little over one in three of all children achieve only 0 cwpm at baseline, compared to fewer than one in five at endline. Furthermore, the cumulative distributions are the most apart at the lower levels, emphasizing that the largest changes happened at the bottom of the distribution. While these visual analyses provide a very informative grasp of distributional differences, it is hard to systematically compare many sub-populations and contexts by individually and graphically analyzing their distributions, and hence the need for more collapsed statistics.

The second key metric shown in Table 3 is the Gini coefficient, which overall seems to behave well. The values observed lie between 0.285 and 0.984. Across all samples and sub-populations, the mean for the Gini coefficient is 0.646 and the standard deviation is 0.221, with values largely lying within a normal distribution. As an interesting benchmark or point of comparison, note that these values are higher than those observed for goods like income. For instance, the latest World Bank data on the national-level income per capita Gini index ranges from 0.242 in Slovenia to 0.630 in South Africa (World Development Indicators). The lowest Gini in Table 1 corresponds to the Philippines in grade 2, when tested in Cebuano at endline, being 0.285. This specific sub-population also has the highest mean ORF. Contrarily, the highest Gini coefficients representing the most unequal sub-populations are each from the Malawi samples, and also correspond to the six highest percent-zero scores across all samples. Interestingly, these subpopulations have extremely low achievement levels. Note that Gini coefficients this high can quickly devolve into “degenerate” metrics as they approach 1, in the sense that they are not very informative (similar to the previous discussion of floor effects). Therefore, measures like the Gini, which are relative to the mean, tend to be less useful as the mean is closer to zero, given how sensitive the metric becomes to changes in the upper tail.^12^ In fact, the issue of degenerate metrics due to prevalence of zeros is related to the fact that the choice of ORF as the main metric may be methodologically unfitting for this sub-sample, but also to the pedagogical implication that ORF is possibly a frontier to cross over for any intervention that aims to raise literacy outcomes in countries similar to those sampled here.

Moving on to the coefficient of variation (CV), this indicator also seems to behave well. The correlation coefficient between the CV and the Gini is 0.81 across all cohorts and samples. Hence, these two measures of inequality move together well, as they did in studies for PRIMR and Tusome (Crouch and Slade, 2020). Across all samples, the mean value of the CV decreases 4.2 to 2.5 from baseline to endline, with a statistically significant decrease in value (p-value of 0.00). Much like the Gini coefficient, the value of the CV is highly dependent on the overall mean. This factor could enter through two different channels. First, if inequality indeed decreases as means increase, then it is also natural to expect the CV to reflect lower levels of inequality given higher means. However, this could also be due to the mathematical fact that the CV is calculated by dividing the standard deviation over the mean. Two populations with the same standard deviation but different means will have different CVs, and in particular the population with the higher mean will have the lowest CV. In this case, all the empirical considerations regarding the expected value of the Gini coefficient conditional on the average seem to also apply to CV, as mathematically they are both computed, either explicitly or implicitly, factoring in the sample mean.

The most problematic metric reported in Table 3 is the ratio of P_x_ to P_y_. In general, a worrying feature of any ratio is how it behaves around values near or at 0. Particularly in the type of foundational literacy data from LMIC that we are using, it is very common for children to score 0. Taking P_90_ to P_10_ as an example, dividing whatever number the 90th percentile is achieving by 0, which is what the 10th percentile tends to achieve, yields a mathematically undefined expression. On the flip side, calculating P_10_ to P_90_ yields a 0 regardless of what the value of P_90_ is—a fully uninformative metric. In fact, for 53 % of subpopulations in our data, the P_90_ to P_10_ cannot be calculated because more than 10 percent of the children assessed recorded a score of 0. While P_75_ to P_25_ can be calculated more frequently, it is available for only approximately 63 % of those sub-populations. Interestingly, across these datasets these ratios are more often undefined for colonial languages such as French and English than for mother tongue languages, partly reflecting the lack of general mastery of these colonial languages by the left tail of the distribution (and a clear disadvantage of early instruction in these languages). In theory, P_x_ to P_y_ ratios could be intuitive metrics due to the simple interpretation that they could yield (e.g. “the Xth percentile performs n times higher than the Yth percentile”). However, this is less relevant as this ratio does not necessarily translate into policy recommendations in the form that “students in the Yth percentile needs n times more resources and instruction as students in the Xth percentile”. Furthermore, unlike measures like the Gini, there are fewer benchmarks or limits that can put into perspective a given value of the P_x_ to P_y_ ratio, making it harder to use as a tool to compare across countries and samples. In general, the nature of foundational literacy data does not make these ratios suitable metrics.

The last key metric displayed in Table 3 is the percent of children reading at 0 correct words per minute (“% zero”). This measure, most closely linked conceptually (in that it is a percentage at a minimum) with “learning poverty” as defined by the World Bank (World Bank, 2019), displays interesting features not captured by the other metrics. First of all, as expected, it “behaves well”, in the sense that it can actually be calculated for all sub-populations. Furthermore, it displays enough variation across sub-samples to make it an interesting point of comparison for different contexts. This metric has a correlation with the Gini coefficient across all of our sub-populations of 0.71, which is high enough to assume that they tend to move together, but also to convey slightly different information. Furthermore, the positive correlation indicates again that the higher the number of low performers in a given sample (as measured by the number of children performing at 0 cwpm), the higher the Gini tends to be. This measure is highly intuitive and actionable, making it easier for policymakers to set learning goals for the bottom of the pyramid. A significant disadvantage of the “percent at zero” metric is that it “dichotomizes” a distribution by splitting it into those above or below an arbitrary threshold. While this is valuable if there is a strong theoretical or empirical motivation for choosing a specific threshold, it could also oversimplify the description of a distribution, perhaps even excluding those just above the selected threshold. Thus, this metric works best when complemented by other measures that also inform about the distribution of skills above the threshold. Note that as result of early observations on the behavior of this indicator, interventions by INGOs and governments started to do two things: a) target a reduction in the percentage of children who could not read at all as a meaningful intermediate benchmark, and b) start to track the percentage of children who would meet a more ambitious but reasonable benchmark such as 30 or 40 correct words per minute (for example, see a goal setting exercise in Ghana described in USAID, 2014). While the World Bank’s target is not to improve the percentage of children who cannot read down to zero, a target related to the percentage of children being below some minimum by age 10 (i.e., out of “learning poverty”), and halving that, is a measure similar to driving to 0 the percentage of children who cannot read at all—but more useful later in the grade structure. The fact that some implementers have found the “% zero” to be a useful benchmark suggests that “% reaching a minimum at age 10″ would also be. But one must make note of the caveats in this paragraph.

Beyond the comparison of the different metrics, Table 3 also highlights some interesting features of the data. A salient pattern is that even within country samples from the same project, different languages have different mean achievement levels and inequality results. As an example of this, the baseline performance in grade 1 in the Philippines ranges from 6.9–21.1, and the Gini coefficient ranges from 0.497 to 0.791. Similarly, the changes from baseline to endline are not uniform. The CV for grade 2 increases in Hiligaynon, decreases in Ilokano, and remains almost constant for Cebuano. These changes are not insignificant, as they may have equity consequences across ethnic groups down the line, were the interventions yielding these changes in inequality to be taken to scale without better understanding these distributional issues. Looking at the data cannot tell us what the precise sociological or pedagogical reasons why this might be—but even merely looking at the data can alert us that there is something that needs to be looked at more substantively. While one hypothesis could be that while part of these differences could be due to differences in how foundational literacy develops across different languages (for examples of this see Spaull, 2016 and Spaull et al., 2020 in South Africa), part of these changes could also be due to differential levels of investment and quality of education across ethnic groups.

### 3.2 The Gini coefficient for oral reading fluency interpreted via Lorenz curves

The Gini coefficient is one of the most well-known measures of inequality, typically used by economists to quantify income or wealth inequality.^13^ Given the prominence of this measure, we present a more detailed discussion of its potential applications and features for the type of data describing foundational literacy. In particular, a nice feature of the Gini coefficient is that it has a visual equivalent through the plotting of “Lorenz curves.” A Lorenz curve is a representation of the cumulative distribution of a certain “good” on the vertical axis (“wealth” when measuring “wealth inequality”, or oral reading fluency, in our illustration of learning measures), graphed against the ordered percentiles of the same good on the horizontal axis. Continuing with the wealth example, any particular point on the Lorenz curve with the coordinates (x, y) shows that all individuals up to the xth percentile on wealth for this sample cumulatively have y percent of the total wealth. Similarly, the Lorenz curve is usually graphed alongside the “line of perfect equality” (i.e., the 45-degree line), which would be the hypothetical Lorenz curve of a population where everyone has the same amount of the good in question. Empirically, the closer the Lorenz curve is to the line of perfect equality, the more equal the underlying distribution is. Visually, the Gini coefficient represents the area between the Lorenz curve and the line of perfect equality as a share of the total area under the line of perfect equality. In this paper, we are treating oral reading fluency, as measured by the number of correct words per minute, as the “good” to be accumulated. We analogize that there is a total amount of “oral reading fluency”, and we study how it is distributed across the population. Of course, ORF is much less of a “zero-sum game” in the short term than goods like wealth, particularly because ORF cannot be redistributed across children the way wealth can. In other words, the only way to change the distribution is by “creating” more of the good, or increasing children’s ORF.^14^

**Fig. 3:**
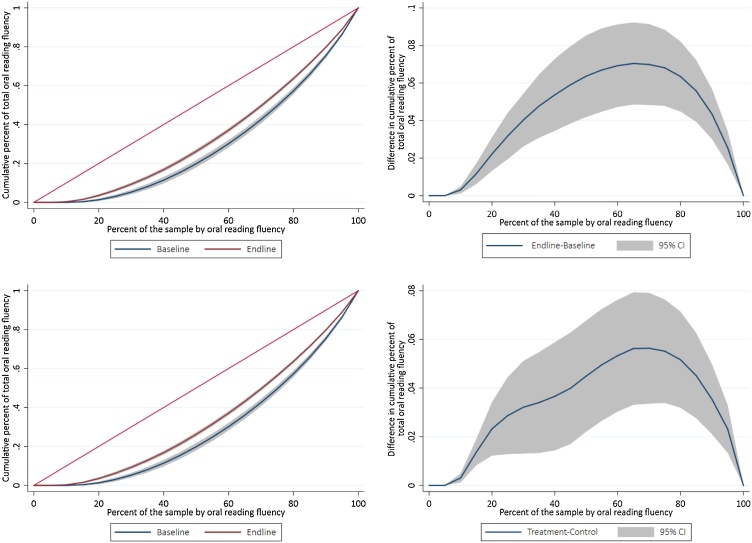
Lorenz curves and contrast plots for the grade 2 experimental sample of the PRIMR intervention in Kenya. **Notes:** the sample for the top row consists of only the treatment group for grade 2, while the sample for the bottom row is both the control and the treatment groups. The outcome used was the oral reading fluency in English. The Gini coefficients for the top row panels (the treatment group at baseline and endline) are 0.419 at baseline and 0.332 at endline. The Gini coefficients for the bottom row panels (the treatment and control groups at end) are 0.332 for the treatment group, and 0.400 for the control group, both at endline.

Given the visual connection between Gini coefficients and the visual representation of Lorenz curves, this metric can be a valuable tool to not only understand distributions better, but the particular differences in these distributions. Furthermore, Lorenz curves allow for the creation of “contrast plots”, as displayed in the panel on the right column of Fig. 3. Contrast plots simply compute the difference between two Lorenz curves by the baseline percentile regardless of the absolute achievement level behind each curve. An additional advantage to Lorenz curves is that both Lorenz curves and contrast plots allow for the computation of confidence intervals which allow for formal testing in differences for a given portion of the distribution across two sub-populations.

We showcase the utility of Lorenz curves and Gini coefficients in analyzing learning inequality and the relationship between inequality and a threshold or “learning poverty” (à la World Bank) concept through two examples. The first example is displayed in Fig. 3, and it uses the grade 2 experimental group from the PRIMR intervention in Kenya. The top row panels of Fig. 3 effectively compare the distribution for the treatment group before and after the intervention, therefore showing what portions of the distribution contributed the most to the improvement in equality for this specific subgroup. A similar interpretation can be given to the bottom row panels: the contrast plot shows what portions of the distributions displayed on the bottom left contributed the most to the improvement in equality for this specific subsample, comparing treatment and control groups. Both of these contrast plots depict a situation in which indeed the underlying distribution became more equal across most of the full range of ability post-intervention.

Neither the shape of the contrast plots, nor their signs at any point of the distribution are mere artifacts of the data. For instance, for the top right panel, the short left-tail of the contrast plot mimics the short, left tail of the Lorenz curves that are precisely at 0 for the cumulative amount of oral reading fluency. This left tail represents the approximately 1 in 10 children that could not read a single word in English at baseline and at endline, and ties to a “learning poverty” threshold concept, but shows the relationship between that and a summary of the distribution such as a Gini. Following the contrast plot, inequality starts to decrease from baseline to endline as percentiles of achievement increase, reaching its maximum around the 65th percentile, where the endline distribution became the most equal compared to the baseline distribution. As mentioned before, it is significant that the contrast plot is always positive, and almost always in a statistically significant manner. It could be that there are cases or countries where it is always negative, or that it is negative in some portions, displaying true heterogeneity in how the distribution changes. In this case, either explicitly or implicitly, the intervention served a wide range of children in this context, and this is something that needs to be examined jointly with the pedagogical features of the program that made this possible.

Importantly, this analysis is not at the individual level, but rather at the distribution level. When we say that the distribution changed at some percentile X, we do not mean that the change happened for the specific child who was at Xth percentile at baseline, but rather for this percentile at baseline and endline, regardless of whether this is the same child or not. This has implications for the analysis of the curves. Consider a hypothetical toy example of an intervention with only 100 children, in which only the median child at baseline (say, Ana) gained from an intervention and that by the endline round, Ana scores higher than the top percentile of the baseline. This change would translate to a contrast plot in the following manner. For the top percentile, it would compare the baseline performance of whatever child was in the top percentile at baseline, to Ana’s endline performance. Similarly, the change at the median would compare Ana’s performance at baseline to the endline performance of whichever child was in the 51st percentile at baseline (as that child now becomes the new median, given Ana’s shift in the distribution). In this sense, this example is meant to reinforce that the Lorenz curve, resulting contrast plots, and Gini coefficients are powerful tools to compare distributions, but not specific individuals’ performance.

**Fig. 4:**
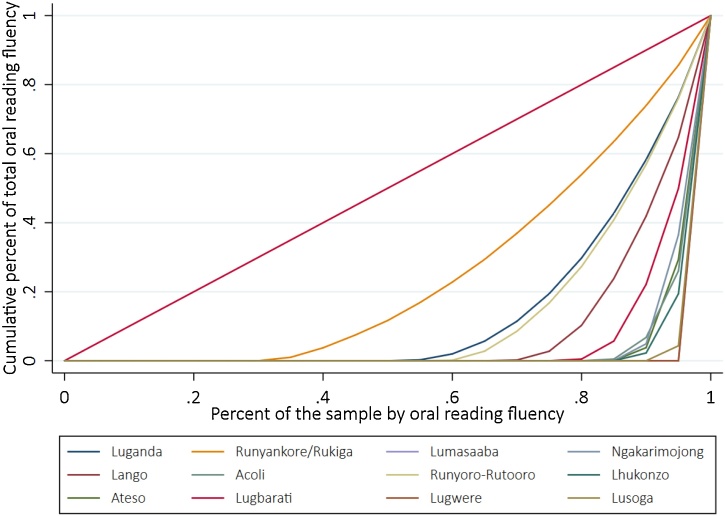
Lorenz curves for oral reading fluency in native language in Uganda. **Notes:** the sub-sample of interest was grade 3 students at baseline. The outcome of interest was oral reading fluency in the mother tongue of each child.

The second insight that can be derived from the visual depiction of Lorenz curves is inter-group comparisons of inequality at a given point of time. Unlike the previous example, this use has less to do with “pure inequality”, and more to do with inequalities across sub-populations linked to their demographic characteristics. To exemplify this approach, Fig. 4 plots the Lorenz curves for the oral reading fluency of a specific sample of grade 3 Ugandan children in their mother tongue. There are stark differences between each of these curves and the line of perfect equality. Languages such as Runyankore-Rukiga or Luganda are relatively closer to the line of perfect equality, with underlying Gini coefficients of 0.679 and 0.737 respectively. Interestingly, Runyankore-Rukiga is a south-western language spoken in the district of origin of the Ugandan president Yoweri Museveni and Luganda is the most commonly-spoken non-colonial language in the capital, Kampala. On the other extreme, languages such as Lhukonzo or Lugwere are much further from the line of perfect equality, with Gini coefficients of 0.897 and 0.949 respectively.^15^ In this sense, inequality, mean performance, and the language of each sub-population within countries are strongly correlated, likely also with other “unobservable” characteristics linked to other types of inequality like political or economic inequality.

**Fig. 5:**
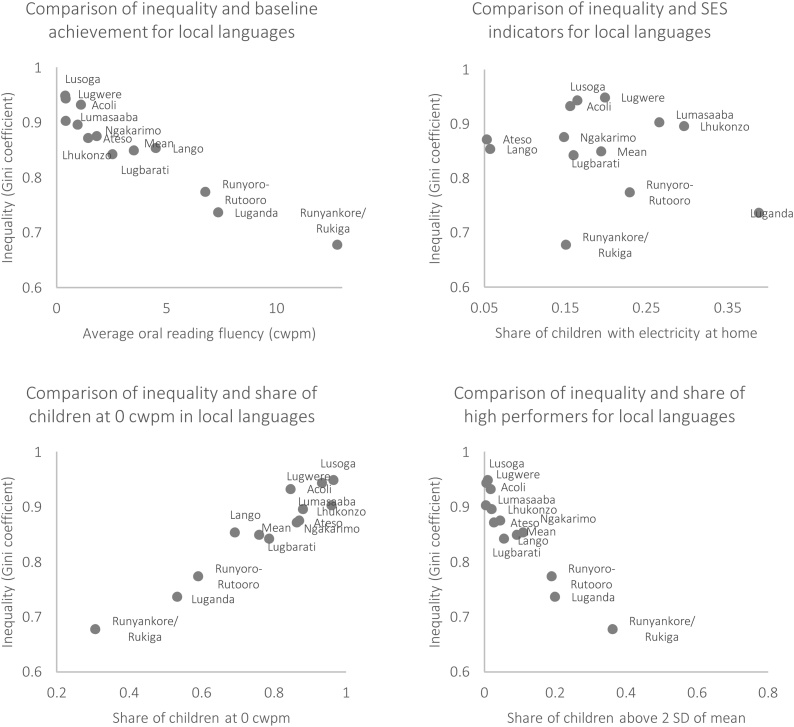
Comparison of Gini coefficient across different Ugandan languages, and different metrics of performance. **Notes:** the sub-sample of interest was grade 3 students at baseline. The outcome of interest was oral reading fluency in the mother tongue of each child. The “share of electricity at home” is defined as the number of children who report having electricity at home divided by the total number of children within each language group. The share of children at 0 correct words per minute (cwpm) is defined as the number of children who could not read any words divided by the total number of children within each language group. The share of children above 2 standard deviations (SD) is defined as the number of children performing at least 2 standard deviations higher than the mean (a threshold of ∼17 cwpm) divided by the total number of children.

In spite of the striking differences in inequality across languages for Ugandan children shown in Fig. 4, one cannot infer the relationship between inequality and average performance from these Lorenz plots. This is an important feature to explore, as high inequality could be driven by a few very high performers even though most children still do well—in a sense, inequality among relatively good performers, which is indeed inequality but less worrisome that a situation driven by fat left tails of children with very low achievement levels. Fig. 5 displays the relationship between the underlying Gini coefficients for each of the Lorenz plots in Fig. 4, and other important variables at the time of describing inequality in outcomes. First, the top left panel shows a negative correlation between Gini coefficient and mean achievement. In other words, in this specific context, as the average child scores higher on oral reading fluency, the overall inequality decreases. This is an interesting result in itself as it sheds some light on the question of whether moving overall means can have effects on inequality, which is not an obvious result in itself. In contrast, the top right panel correlates the Gini coefficient with an indicator of socioeconomic status like household level access to electricity. Indeed, the relationship is much weaker than the correlation between inequality and mean outcome. In this sense, we see that to move unequal results, targeting performance can be a more proximate input to the desired outcome than interventions targeted based on the socioeconomic status of children.

Another interesting result that stems from Fig. 5 is the high correlation between the Gini, and the presence of outliers in either end of the distribution. On the bottom row, we show that a lower Gini is indeed strongly correlated with lower share of low performers (measured as the percent of students at 0 cwpm) and higher shares of high performers (measured as the percent of students above two standard deviations of the mean). The Lorenz curves shown in Fig. 4 are characterized by long left tails (or rather an intercept with the horizontal axis), with many children reading 0 correct words per minute. That is, the correlation between lower shares of low-performers and the Gini coefficients is not surprising, as pushing kids from the left-tails towards higher levels should indeed result in more equitable distributions. The fact that a lower Gini is correlated with larger shares of high performers is perhaps more surprising in isolation, but not in this context. A larger number of high performers, as we are measuring it, is a reflection of an underlying sub-distribution that is more centered towards the right (i.e. higher mean), and hence a larger proportion of the right tail makes the cut for “higher performers.”

From these two correlations, one could hypothesize that targeting either high or low performers might decrease inequality at the same rate, but this argument would miss two key points. First, as previously discussed, a larger number of high performers is just an artifact of where each language distribution is generally placed, not of a special group of high performing students. Furthermore, targeting an intervention only at high-performing students (however they might be defined in different contexts), without moving the rest of the distribution, is likely to increase inequality instead. The second key issue is that the share of low-performing students is much higher than the share of high-performing students for all languages. Even languages with relatively high means such as Luganda have 53 % of students performing at 0 cwpm, and have only 20 % as “high performers.” Other extreme cases such as Lugwere have 96 % of students at 0 cwpm and 0% at the high-performing echelons. Therefore, inequality-reducing policies that target the critically-low performing students are likely to find a much larger audience than those aiming at relatively high-performing students. Mathematically, policy targeting mean performance of the higher-performing students would need to be several times more effective at moving the mean than its counterpart of targeting low-performing students to move the overall average by the same amount.

## 4 How is inequality distributed within countries?

Beyond the different determinants of inequality within certain language, country, or grade subgroups, it is also critical to understand how inequality is distributed within a country. In the particular case of education, inequality can be clustered around a “natural unit” of education delivery among the population: schools. This is a key empirical question particularly for the bottom of the pyramid, as it is pivotal to an understanding of whether the low performing students are concentrated in certain schools or whether it is widespread across the whole geographic area covered by the data. In fact, previous work such as Brunner et al. (2018) has revealed that the between-school vs. within-school variation in learning, but also other measures like motivation or affect, can greatly differ by country. To explore this question in our context, we will follow a simple variance decomposition of the kind that is typically applied to TIMSS or PIRLS data. We base our approach on the method proposed by Foy (2005). In particular, we will understand the “between-school” variance as the intraclass correlation (ICC) within schools (a measure more familiarly used in determining ideal sample size in clustered random sampling, but useful also in this context), which is the between-school variance as the proportion of the sum of the two variance components, described in the following equation (Foy, 2005):

$$ICC=\frac{\sigma_{B}^{2}}{\sigma_{B}^{2}+\sigma_{W}^{2}}$$

Where $\sigma_{B}^{2}$ is the between-school variance, and $\sigma_{W}^{2}$ is the within-school variance. This approach is particularly enticing given the straightforward interpretation of the outcome: in this case, ICC is always between 0 and 1, and it represents the share of the total variance in outcome (oral reading fluency) than can be explained by differences between schools. Furthermore, very similar approaches have been implemented in reports for international tests such as PISA (OECD, 2017), allowing us to compare our results with the results found in typically more developed countries (and much later grades).^16^ We display the variance decomposition for different data sets in Table 4 below, by testing round. One way to interpret these numbers is that the higher the values in the “between” columns, the more concentrated oral reading fluency is within certain schools, as differences in the outcome would be largely explained by differences between schools.

**Table 4:** variance decomposition of oral reading fluency between/within schools.

Country	Language	Grade	Baseline		Endline	
			Between	Within	Between	Within
Malawi	English	1	11 %	89 %	13 %	87 %
		3	18 %	82 %	17 %	83 %
DRC	Tshiluba	3	19 %	81 %	24 %	76 %
	Kiswahili	3	36 %	64 %	12 %	88 %
	Lingala	3	34 %	66 %	19 %	81 %
	French	4	20 %	80 %	28 %	72 %
	French	5	32 %	68 %	27 %	73 %
	French	6	31 %	69 %	43 %	57 %
Egypt	Arabic	2	35 %	65 %	.	.
Kenya PRIMR	English	1	34 %	66 %	47 %	53 %
		2	39 %	61 %	43 %	57 %
	Kiswahili	1	27 %	73 %	29 %	71 %
		2	32 %	68 %	31 %	69 %
Philippines	Cebuano	2	38%	62 %	26 %	74 %
	Ilokano	2	36 %	64 %	36 %	64 %
	Hiligaynon	2	33 %	67 %	42 %	58 %
	Maguindanaoan	2	31 %	69 %	33 %	67 %
Uganda	English	1	8%	92 %	35 %	65 %
		2	40%	60 %	39 %	61 %
		3	39 %	61 %	31 %	69 %
		4	37%	63 %	35 %	65 %
		5	37%	63 %	35 %	65 %
	AcolI	1	4%	96 %	9%	91 %
		2	19 %	81 %	19 %	81 %
		3	13 %	87 %	19 %	81 %
		4	11 %	89 %	.	.
		5	25 %	75 %	.	.
	Luganda	1	6%	94 %	25 %	75 %
		2	36 %	64 %	17 %	83 %
		3	22%	78 %	13 %	87 %
		4	10 %	90 %	11 %	89 %
		5	21 %	79 %	.	.

Table 4 displays three interesting patterns.^17^ Firstly, the between-school variance in foundational literacy in LMIC has a wide range. The ICC for English in the Malawi sample for grade 1 was only 11 %, while it was 34 % for the Kenyan sample also in grade 1, also tested in English. In other words, performance is much more clustered by schools in the Kenya sample than in the Malawi sample (again, at least for these two samples). The second interesting feature in Table 3 is the typically low between-school variation for grade 1 across contexts, with the exception of Kenya. In countries where preschool penetration is low and uncommon even among the relatively wealthy sub-groups, it is natural to expect that children across all schools enter the first grade with close to no oral reading fluency. This means that the low between-school variation is due to the fact that there are many children at 0 during the baseline round, the modal experience across all schools. This is typically reverted by the endline, where Uganda provides a stark contrast: first grade Luganda goes from an ICC of 6%–25%, and English from 8% to 35 %. Contextualizing this change, in the 2015 OECD PISA data only Iceland had an ICC of less than 8%, and only 3 countries out of 35 OECD countries had IC higher than 35 %. In other words, achievement in Uganda goes from being as little clustered as the least concentrated country in the OECD to as clustered as the most concentrated countries in the OECD *in just one year of schooling*.

The last pattern, or lack of thereof, is the similarity in between-school variance by language tested. In the DRC sample, the range for French, Kiswahili and Lingala is very similar, Tshiluba being somewhat lower than these three. In Kenya, the between-school variation is very similar for Kiswahili and English. The Uganda sample is the exception, where between-school correlation is much higher across grades for English than Luganda. The context of each intervention clearly matters: students were only tested in Luganda if that was their mother tongue, whereas they were all tested in English. As English is a skill learned at school more than at home, one could expect a larger role of schools in this number, particularly as the sample is expanded from only Luganda-speaking regions to all of Uganda.^18^

More broadly, policy recommendations may vary by the degree of clustering of inequality. That is, the policy recommendations (e.g., a broader intervention targeting most schools) for a country with overall low learning levels and low between-school variation would be very different from a country with overall low learning levels and high school-to-school variation, suggesting that some schools are driving the low performance (and the resulting intervention would likely be more targeted towards these low performing schools). In other cases where one might observe low levels of average performance but low between-school variation, ability grouping for remediation might make the most sense. Understanding the concentration of performance across different clusters, as we have displayed here with schools, is a pivotal step in the process of designing interventions aimed at improving both average performance and reducing inequality.

## 5 What role does socioeconomic status play in inequality and changes in inequality?

**Fig. A2:**
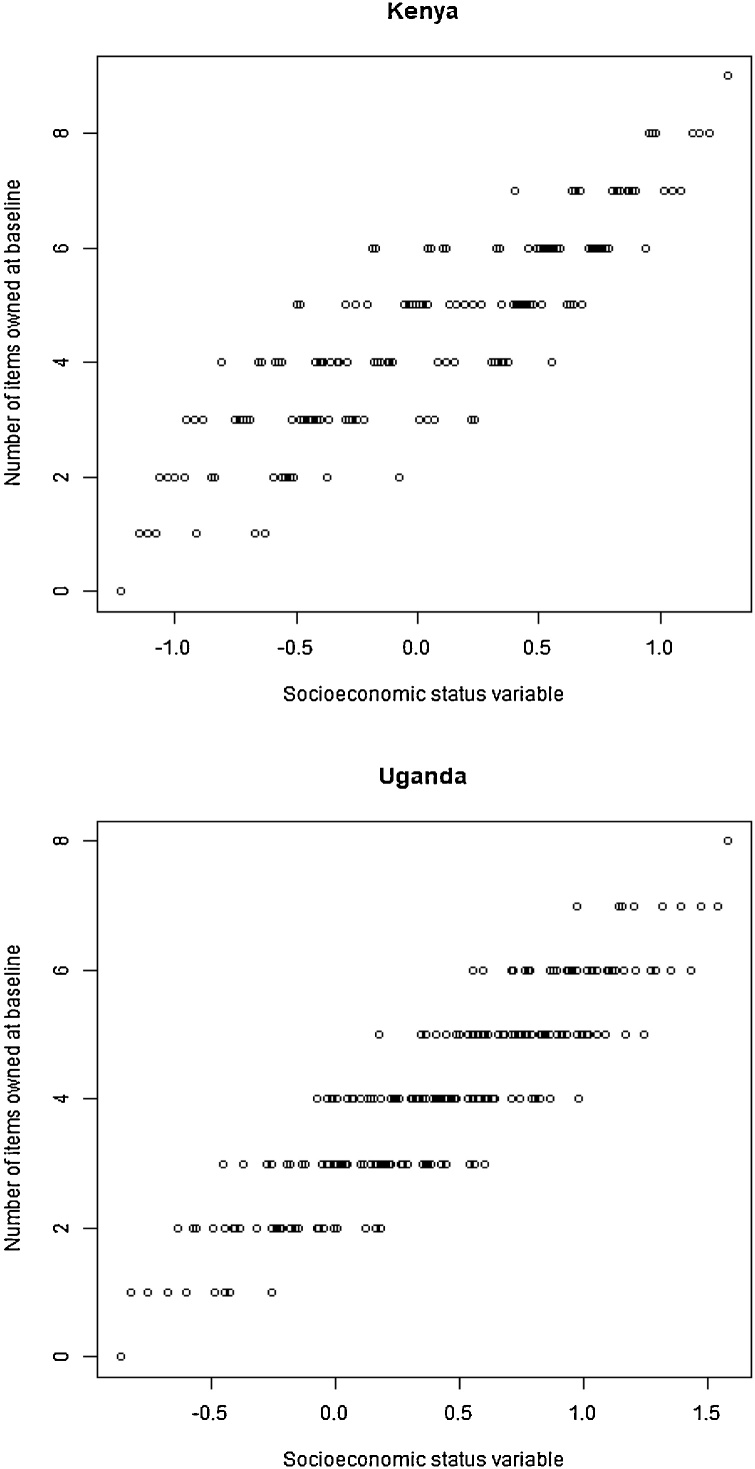
Comparison of socioeconomic index assigned through principal component analysis, and number of items owned, by country.

Moving away from analyses related to “pure inequality”, we also use our main metrics to explore inequality from the lens of socioeconomic status. In order to do this, we leverage the two country samples for which we have reliable data on socioeconomic status: Kenya and Uganda. Children in all of these countries were asked if their household possessed each of seven different assets (e.g., a T.V., a radio, or a motorcycle). Using these responses, we perform a principal component analysis to yield a single variable which encapsulates each child’s socioeconomic status (SES). The relationship between the number of assets owned and the socioeconomic status variable assigned is shown in the appendix in Fig. A2. As expected, socioeconomic status is highly correlated with literacy achievement. We observe that an increase of 1 standard deviation (SD) in SES is correlated with a 0.31 SD statistically significant increase in oral reading fluency in the Kenyan sample and 0.20 SD in the Ugandan sample.^19^ Subsequently, we compute some of the metrics discussed in previous sections, but by socioeconomic quartile to understand how these measures stand within socioeconomic groups, and how they evolve throughout time. These results are displayed in Table 5 below.

**Table 5:** Comparison of different inequality metrics at baseline and endline by socioeconomic status quartile.

Inequality measure	SES Quartile	Kenya (PRIMR)						Uganda					
		Grade 1			Grade 2			Grade 1			Grade 2		
		BL	EL	EL-BL	BL	EL	EL-BL	BL	EL	EL-BL	BL	EL	EL-BL
Gini	1	0.72	0.64	−0.07	0.51	0.45	−0.06	0.92	0.92	0.01	0.824	0.697	−0.13
	2	0.55	0.50	−0.05	0.40	0.39	−0.01	0.87	0.88	0.02	0.728	0.713	−0.02
	3	0.46	0.46	0.00	0.34	0.31	−0.03	0.91	0.90	0.00	0.78	0.729	−0.05
	4	0.44	0.41	−0.02	0.32	0.28	−0.05	0.91	0.85	−0.06	0.626	0.693	0.07
	All	0.53	0.50	−0.04	0.40	0.35	−0.05	0.90	0.88	−0.02	0.747	0.707	−0.04
CV	1	1.5	1.2	−0.2	0.9	0.8	−0.1	3.2	3.2	0.0	2.2	1.4	−0.7
	2	1.0	0.9	−0.1	0.7	0.7	0.0	2.3	2.6	0.2	1.6	1.5	−0.1
	3	0.8	0.8	0.0	0.6	0.5	−0.1	3.2	2.9	−0.2	1.8	1.6	−0.3
	4	0.8	0.7	0.0	0.6	0.5	−0.1	3.6	2.3	−1.3	1.2	1.4	0.1
	All	1.0	0.9	−0.1	0.7	0.6	−0.1	3.2	2.7	−0.5	1.7	1.5	−0.2
0%	1	0.46	0.34	−0.12	0.19	0.15	−0.05	0.80	0.82	0.02	0.658	0.447	−0.21
	2	0.25	0.20	−0.05	0.11	0.10	−0.01	0.75	0.81	0.06	0.511	0.467	−0.04
	3	0.16	0.14	−0.02	0.06	0.06	0.01	0.78	0.81	0.03	0.584	0.517	−0.07
	4	0.14	0.12	−0.01	0.03	0.02	−0.01	0.78	0.74	−0.04	0.357	0.459	0.10
	All	0.24	0.19	−0.05	0.09	0.08	−0.02	0.78	0.78	0.01	0.533	0.475	−0.06
MRF	1	12.8	20.8	8.0	30.5	45.3	14.8	1.1	2.9	1.7	3.7	11.2	7.5
	2	26.8	32.5	5.6	45.7	54.2	8.5	2.1	3.3	1.1	6.7	11.9	5.2
	3	29.4	37.4	8.1	55.0	63.0	8.0	1.5	2.6	1.1	4.9	10.3	5.4
	4	35.2	37.2	2.0	63.1	66.6	3.5	1.4	6.6	5.2	12.1	9.3	−2.8
	All	26.6	32.6	5.9	49.4	58.1	8.7	1.5	4.3	2.8	6.7	10.7	4.0

**Notes:** abbreviations used in the table explained further here. "BL": baseline; "EL": endline, "EL-BL": difference between endline and baseline; "SES": socioeconomic status. Mean reading fluency (MRF) measured as the average cwpm across all children in each quartile. Socioeconomic status quartiles were calculated separately for each country.

**Fig. 6:**
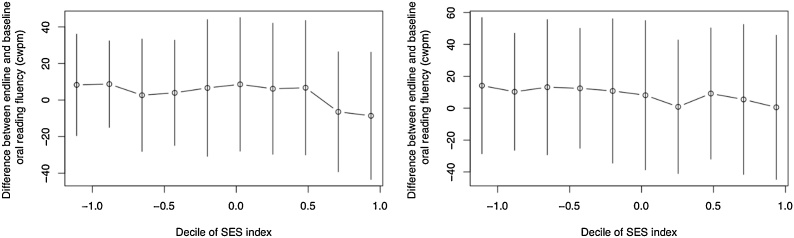
Changes in oral reading fluency from endline to baseline in Kenya PRIMR, by grade and socioeconomic status. **Notes:** the left panel shows grade 1 in Kenya (PRIMR), and the right panel shows grade 2 in Kenya (PRIMR). The confidence intervals correspond to one standard deviation above and below the conditional mean at each decile.

Table 5 displays several interesting features. As expected, median reading fluency increases with SES quartile, while the percent of children at 0 cwpm decreases with SES quartile. The fact that even higher quartiles have non-zero values for this last metric simply shows how pervasive the prevalence of zero scores is: performance can be very poor even among the relatively well-off in these countries. When we observe measures of inequality such as the Gini, we see that lower quartiles tend to be more unequal “within themselves” than higher quartiles. For instance, grade 2 at baseline for the first quartile has values of 0.51 in Kenya, and 0.82 in Uganda, whereas the fourth quartile has values of 0.28, and 0.63 respectively. This pattern mimics a familiar pattern described in previous sections: as the mean increases, the Gini tends to decrease.^20^ This pattern is also followed by the coefficient of variation, which is lower for higher quartiles. Of special notice is the fact that these programs reduced inequality across the board, even though they were not aimed at inequality, only at foundational skills.^21^ More broadly, higher heterogeneity within the poor compared to their wealthier counterparts is a phenomenon observed in other data sets, for example in World Bank (2007), where Peruvian data displays wider variance in achievement for the poorest groups. This “variation in the variation”, or heteroskedasticity, is indeed indicative that the bottom of the pyramid is a more diverse set of students than more advantaged groups, reinforcing the need for greater attention to their achievement levels and the distribution of achievement. The World Bank (2007) notes for instance that in Peru, a very heterogenous country, there is, among the poor, much more “within” linguistic and nutritional variation than there is among the less poor. Finally, we see that the changes in inequality from baseline to endline do depend heavily on the context. While the Kenyan sample does not seem to have much heterogeneity in this respect, the Ugandan sample does. To further reinforce the small variation in changes across testing rounds, across socioeconomic groups in Kenya, Fig. 6 below shows the change in oral reading fluency by socioeconomic status deciles. There is a slight decreasing trend in both grades (meaning that poorer children benefited slightly more), but not enough to claim with certainty that this is a marked pattern. Indeed, the differences in how inequality evolved across rounds for each country reinforces the importance of exploring distributional changes within interventions.

## 6 Limitations

The fundamental purpose of this paper is to explore the methodological alternatives that educational systems, policymakers, and researchers have at their disposal to track levels and changes in the inequality of learning outcomes. Beyond being just an empirical exercise, these metrics of inequality can shed light on the educational and policy implications to address inequality, especially among the most disadvantaged populations around the world. As such, there are several limitations in the reach and depth of some of the claims we can make. First of all, this paper showcases how some of the metrics discussed can be calculated for sub-populations of interest. It does not provide updated, nationally-representative statistics about learning inequality levels within each of the countries explored. Secondly, we do not argue that oral reading fluency should be the “gold-standard metric” to compute inequality metrics. As we discuss in previous sections, our choice of ORF was based rather on the need to pick a single measure to compute these indicators for all six of our samples. The metric to compute inequality indicators should be carefully tailored to the context, with considerations about how floor and ceiling effects can come into play at the intersection of the difficulty of the metric and population of interest. The presence of floor (or ceiling) effects, and the poor performance of inequality metrics that comes with these, is particularly likely to occur if a large share of the students tests at zero percent (or 100 percent) in the skill chosen to estimate these metrics. A similar argument can be made about the choice of EGRA as the assessment tool: our argument is not that it is the ultimate tool for the purposes of quantifying inequality, but rather that it is an assessment (out of probably several) which records indicators with desirable properties for the estimation of these metrics. These limitations highlight the fact that, as we mention before, all of these metrics are flawed in their own way. Taken individually, they each provide an incomplete picture of learning inequality, and hence, a complete assessment of learning inequality needs to leverage the complementary features in all of these metrics.

Finally, an additional limitation of these analyses is that the benchmark for the inequality metrics and even the intra-cluster correlations are likely to vary depending on the language transparency, linguistic structure, and orthography of each specific language, making it harder to produce cross-context comparisons—but that is not the point. Taken together, these limitations are important to consider but should not distract from our call for more empirical and policy attention on learning inequality, beyond averages, especially through the methods proposed here. In all, we realize that this type of analysis is new to economists and educationists, and as such, what is causing the improvement in inequality may not be clear. The paper is not suggesting that these metrics should be monitoring indicators to *all* projects. It would be unreasonable to suggest the interventions monitor these kinds of indicators at all points in time and in real time, but rather, that these measures should be seen as guidelines for the design, methods, and overall approach for foundational learning projects that do have an element of inequality-reducing efforts at their core.

## 7 What are the possible pedagogical and policy implications?

Identifying the precise drivers of the observed inequality reductions is a complex task, and one for which our current data does not allow a clear disentangling of competing hypotheses. None of the programs which generated the data used in this paper specifically targeted inequality reduction, nor were they designed to study distributional features and changes in the population of interest. Therefore, the best we can offer at this point is informed speculation—speculation which can drive hypotheses for further research that is more deeply pedagogical than this data-oriented paper, regarding what causes and shapes inequality at the bottom of the pyramid. Having said this, the application of our analytic methods to the presented data has shown several interesting patterns which may inform a more thorough discussion on learning inequality.

First of all, we have seen that even the groups of lowest-performing and poorest students have variation in performance. While overall distributions are generally characterized by long (or “fat”) tails of children who score 0 correct words per minutes or below some level such as level 1 in TIMSS or PIRLS, there often more variation in performance among the poor than among their better-off counterparts. A hypothesis consistent with this pattern is that inequality in learning outcomes could be closely tied to the presence, or lack of, system-wide and enforced standards around learning (Atuhurra and Kaffenberger, 2020). Standards that are coherent across a system and aligned to the right instructional level could improve system-wide accountability for the lowest performing portion of the distribution, hence reducing both inequality and the observed heteroskedasticity. In a sense, the fact that poorer students have more variance in achievement compared to wealthier students, as we document here and in other work such as World Bank (2007), can be interpreted as the better-off groups being more effective at inducing a more standardized delivery of education for their children. We do not know for certain if this is why we observe this pattern of heteroskedasticity, but the issue of asymmetrical political forces across different socioeconomic groups shaping educational delivery could benefit from future analysis. Issues such as ethnic affinity between policymakers and specific regions have been documented as drivers of provision of public goods (Ejdemyr et al., 2017), but less is known about whether different political forces can also affect the *standardization* of the quality of these public goods, in particular learning outcomes and their distribution within a country.

Another interesting pattern found was a clear trade-off in the interpretability of certain metrics, and how much information about the full distribution of literacy skills they each convey. While a deprivation metric like “learning poverty” might be helpful for general goal-setting of different policies, there is enough variation above and below this threshold that it is worth understanding what the full distribution looks like. Conversely, a metric like the Gini coefficient might not have a “natural” interpretation, but it conveys more information about the shape of the distribution than “percent of children at 0 cwpm.” In this sense, studying inequality solely through the lens of one metric, or one type of inequality (i.e., solely through “pure” inequality, or solely through the socioeconomic lens) might miss an important portion of the children that require speedy policy attention. This is reinforced by the fact that there seems to be more learning inequality in the more disadvantaged socioeconomic groups than in wealthier subgroups, highlighting the need to take a serious, and comprehensive look at the needs and disaggregate achievement levels at the bottom of the pyramid.

Similarly, we find evidence that as the mean literacy level increases, inequality tends to also decrease. Other studies such as Crouch and Slade (2020); Crouch and Rolleston (2017), and Crouch and Gustafsson. (2018) have found a similar relationship between inequality and average performance. This is neither trivial nor a necessity of growth. The fact that increasing mean scores at the very low levels of performance could yield more equitable outcomes is highly relevant for how policy can tackle both of these issues at once. On the one hand, this could be simply an unintentional by-product of programs like PRIMR and Tusome, which as Piper et al. (2016)note: “Although the project [PRIMR] did not explicitly target the [income] poor, the basic strategies in teaching literacy and numeracy skills have proven to be effective in supporting pupils at risk for reading difficulties. PRIMR is organized in ways that align with how best to support those at risk” (p. 72.). Therefore, specific design features of programs might be targeting inequality implicitly by choosing to provide instructional or other types of support for the lowest performing students. On the other hand, this relationship could be a reflection that mathematically, metrics such as the Gini coefficient or the coefficient of variation are mean-specific, so two distributions with the same dispersion but different means will perform differently on these measures. However, a key question is whether this mathematical feature misrepresents the reality on the ground.

Precisely on this point, foundational literacy development, and more broadly the development of foundational skills, does not need to be a linear process. In other words, it does not need to be the case that getting a child from 0 to 20 cwpm takes the same resources and time as getting them from 60 to 80 cwpm. In fact, it is very likely that children face different binding constraints for growth throughout their foundational literacy development. For instance, Glewwe et al. (2009), find that providing textbooks of higher quality was only relieving a binding constraint for students that were at a high enough English level to truly benefit from these inputs. A similar argument can be made about Teach at the Right Level-type of interventions: high performers were more likely to keep up with the instruction pre-intervention, so the binding constraint relieved by TaRL was more prevalent in lower performing students (Banerjee et al., 2017). As mentioned before, the constraints for reading the first word that takes a child from 0 to a few (say, 10) cwpm requires basic alphabetic knowledge, and subsequent phonic translation of these letters into sounds and syllables, which can be strung together to form a word. On the other hand, getting a child to read their 81st word per minute may take an increase in familiarity with more complex syllables, longer words, or even just additional practice to read more fluently. As with other cognitive processes, it is likely that there are diminishing returns to efforts at improving reading fluency, and that growth slows down the more fluent in reading children become. Still, learning is a dynamic process, and diminishing returns in fluency might not imply diminishing returns in learning more broadly, as returns also depend on the outcomes of interest. If an intervention focuses on foundational skills, and the outcomes measured are also foundational skills, then it is likely that already high-performing students will display diminishing returns to how much they can gain. If on the other hand the outcomes measured are (for example) science grades, where foundational skills are necessary but not sufficient, then the role of diminishing returns in the outcome may not be as large—but the absolute effort required might be higher.

The pressing importance of studying inequality in learning outcomes is currently being heightened due to the ongoing COVID-19 crisis. Although at the time of preparing this manuscript, it is too soon to have precise data on learning and enrollment losses in LMIC, educational researchers have made informed hypotheses about the negative and asymmetrical effects of the pandemic on education in LMIC. For example, we know from the Ebola crisis in Sierra Leone that school closures led to persistent drops in enrollment of up to 17 percentage points for young girls in the absence of any intervention (Bandiera et al., 2020). This kind of shock to the system can revert years of progress both in terms of average enrollment, and in enrollment of historically disadvantaged groups. Furthermore, calculations by Kaffenberger (2020) estimate that by 2030, the cohort that was in first grade in 2020 in an average low-income country would have lost a full year of learning by the time they get to grade 10 as a result of the pandemic. Worryingly, these learning losses in LMIC are likely to happen very unequally across socioeconomic groups. Better-off students may have access to reading materials at home, educated relatives, and technology that the poorest children are likely to be completely deprived during school closures. In countries like Mexico or Peru, 94 % of households in the top income quintile have access to computers at home, while less than 10 % of all households in bottom income quintile do (Rieble et al., 2020). These serious gaps in how prepared different households were to manage student engagement during school closures are likely to have lasting impacts on schooling, and are yet another reason to place more focus on inequality analyses of learning outcomes and on the foundational skills, which tend to decay if unattended.

One last implication is based on the narrower results of the paper itself: it seems useful to measure inequality changes (and baselines), not just changes in the average learning scores. However, at least with metrics such as ORF (and changes in ORF) that may have a lot of zeroes, the interpretations of these measures will be more insight-generating if the interpretation is always in light of the percentages of children at zero and the mean performance. Since these two is something that most evaluators already do, at least for foundational learning programs, it seems an easy thing to implement. One may the simply need to add some of the inequality measures (as in Table 3), and/or examine the whole distribution (as in Fig. 2).

In light of the results presented in this paper, future design and evaluation of policies needs to have a keen eye on inequality. Either implicitly or explicitly, through action or inaction, educational policies always take a stance on how they shape inequality. A coherent educational system needs to be aligned to properly cater, sort, and nurture the learning process of all students. This paper has illustrated metrics and styles of analyses that could be useful in informing policies and practices aimed at improving the distribution of learning results.

## Author contributions

Rodriguez-Segura: Conceptualization; Formal analysis; Methodology; Validation; Visualization; Writing and review and editing.

Campton: Data curation; Formal analysis; Methodology; Some writing and review and editing.

Crouch: Conceptualization; Funding acquisition; Methodology; Project administration; Resources; Supervision; Writing and review and editing.

Slade: Conceptualization; Data curation; Project administration; Some writing and review and editing

## Support

DFID (now FCDO) and 10.13039/501100000996DFAT, via the RISE Programme, provided partial writing support. 10.13039/100000200USAID provided most of the support to original data gathering for uses not related to this paper; data uses for this paper are entirely secondary to original purpose. Other support was through the authors’ normal employment or through personal effort. Supporters played no intellectual role in research design or execution.

## Authors’ note

The authors would like to thank the RISE Programme for partial support in the preparation of this study. Abbreviations frequently used: “SES”- socioeconomic status; “cwpm”- correct words per minute, “ORF” - oral reading fluency. Throughout this paper and for each sub-group, “baseline” will refer to the first round of data collected, and “endline” to the last round of data collected, regardless of whether these coincide with the empirical definitions of baseline and endline by study of origin for each dataset. All calculations made by authors unless otherwise stated.

## Declaration of Competing Interest

Authors declare they have no conflict of interest related to the writing of this paper or the findings presented.

## Figures and Tables

**Fig. A1:**
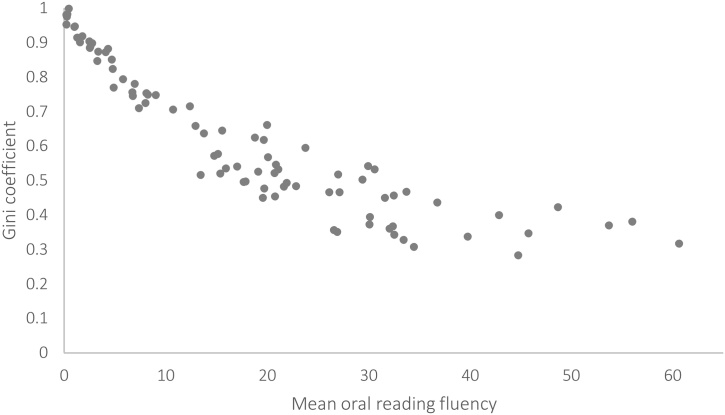
Comparison of Gini coefficient and mean oral fluency for all subpopulations analyzed for Table 3. **Notes:** each dot represents the Gini coefficient and the mean oral reading fluency (as measured by correct words per minute) for different subpopulations across all data sets used.

**Table A1:** Variance decomposition of % children at 0 cwpm between/within schools.

Country	Language	Grade	Baseline		Endline	
			Between	Within	Between	Within
Malawi	English	1	17 %	83 %	15 %	85 %
		3	16 %	84 %	17 %	83 %
DRC	Tshiluba	3	28 %	72 %	19 %	81 %
	Kiswahili	3	37%	63 %	21 %	79 %
	Lingala	3	43 %	57 %	19 %	81 %
	French	4	25 %	75 %	28 %	72 %
	French	5	34 %	66 %	26 %	74 %
	French	6	29 %	71 %	18 %	82 %
Egypt	Arabic	2	29 %	71 %	.	.
Kenya PRIMR	English	1	35 %	65 %	34 %	66 %
		2	30%	70 %	25 %	75 %
	Kiswahili	1	28 %	72 %	31 %	69 %
		2	28 %	72 %	18 %	82 %
Philippines	Cebuano	2	18 %	82 %	16 %	84 %
	Ilokano	2	25 %	75 %	24 %	76 %
	Hiligaynon	2	25 %	75 %	35 %	65 %
	Maguindanaoan	2	32 %	68 %	31 %	69 %
Uganda	English	1	8%	92 %	32 %	68 %
		2	33 %	67 %	33 %	67 %
		3	28 %	72 %	35 %	65 %
		4	25 %	75 %	20 %	80 %
		5	19 %	81 %	13 %	87 %
	AcolI	1	8%	92 %	9%	91 %
		2	31 %	69 %	19 %	81 %
		3	10 %	90 %	25 %	75 %
		4	16 %	84 %	.	.
		5	19 %	81 %	.	.
	Luganda	1	6%	94 %	24 %	76 %
		2	32 %	68 %	12 %	88 %
		3	20 %	80 %	12 %	88 %
		4	11 %	89 %	8%	92 %
		5	14 %	86 %	.	.

## Footnotes

1 As an interesting example of this emphasis within the SDG 4, nouns denoting a sense of equality are used 13 times in the main policy document.

2 These tests emerged partly from Dan Wagner’s influential 2003 paper (Wagner, 2003), in which he called for “smaller, quicker, cheaper” (SQC for short) measurements. He later went on to discuss these in further detail in Wagner (2011) and Wagner (2013).

3 People’s Action for Learning Network assessments, informally known as Citizen-Led Assessments, Early Grade Reading Assessments as described by Dubeck and Gove. (2015).

4 The percent of children scoring at zero is not strictly a measure of inequality, but rather the value of the cumulative distribution function at 0 (or any other arbitrary, but meaningful threshold). In fact, this measure is more closely linked to “deprivation” measures, which are in spirit similar to measures of poverty using a poverty line (i.e., an arbitrary threshold much like zero correct words per minute) However, this measure is strongly shaped by the distributional properties of samples, especially when students are as concentrated on the lower end of the distributions as they are in terms of learning in many LMIC. Due to its closeness to inequality in terms of measurement and spirit of the metric, we decide to also include it.

5 See https://earlygradereadingbarometer.org/pages/about_egra for a description of EGRA, a listing of all more or less “official” applications, and see to the main results in all those applications.

6 One application of EGRA used in one of the interventions for which we use the data is described in Piper, King, and Mugenda (2016) for Kenya, and can be used to give a flavor for the psychometric properties of the assessment. In Kenya, the version of EGRA used had a Cronbach’s alpha of 0.86 for English and 0.90 for Kiswahili, with item-test correlations for ORF of 0.87 and 0.90 respectively. An ORF task with a mean of 40 cwpm would typically have had a standard error of 1.3 or so, thus allowing tight confidence intervals given sample sizes of about 2000. Generalizing across 9 countries, we note that Gove et al. (2015) show models explaining student success using EGRA in a wide range of countries had p values averaging 0.008, suggesting that the tool’s summary measures are well-correlated with the normal educational factors such as whether the child had repeated the grade being tested (negatively correlated as is usual) and sociological factors such as whether the students have books at home (positively correlated as is usual).

7 While there is no theoretical maximum for this metric, for all practical purposes scores above 200 (roughly 3.5 words per second) are exceedingly rare in the contexts where EGRA is typically applied.

8 The correlation between the [English-language] reading fluency and comprehension subtasks on the Stanford Achievement Test was 0.91. Comparable correlations between fluency and comprehension have been found for assessments conducted in numerous other languages. (Bulat et al., 2014; LaTowsky et al., 2013; Management Systems International (MSI), 2014; Pouezevara et al., 2012, etc.) Analyses similar to those presented in this paper can also be conducted using measures from EGRA’s other fluency-based subtasks (e.g., *correct letters per minute*, *correct syllable sounds per minute*, *correct nonwords per minute,* etc.). For further detail regarding EGRA, the design of various fluency subtasks, and the validity of the constructs, see the EGRA Toolkit 2.0, RTI International (2015).

9 There is a substantial literature debating the merits and shortcomings of EGRA data that we will not revisit here, as it falls outside of the scope of our topic.

10 Even though we do compute these metrics for all these sub-populations, this would make the table much longer and cumbersome to interpret. Therefore, we explicitly chose sub-populations that illustrate certain points we will make about the performance of different metrics, but did not “cherry-pick” rows to favor one interpretation over another, or much less to give preference to one policy recommendation over another.

11 This is especially true since metrics such as the Gini coefficient or the coefficient of variation (CV) are relative to the distribution’s mean. For example, consider two populations. The first population (n=10) has 50% of individuals at 1 cwpm, and 50% at 2 cwpm. The second population (n=10) has 50% of individuals at 40 cwpm and 50% at 41 cwpm. Both of these populations would have the same variance, but very different Gini and CVs. The Gini coefficient for the first population is 0.167 and for the second population is 0.006, while the CV for the first population would be 0.33 and 0.01 for the second population. It is then empirically and intuitively clear that the former is much more unequal.

12 For a comparison of mean scores and Gini coefficients of all sub-populations tested in Table 3, see Fig. A1 in the appendix.

13 Another use in the field of education is demonstrated by Thomas et al. (2003), who estimate the Gini coefficient for a panel of countries, based on the years of schooling (not learning).

14 This is under the assumption that children cannot be made to *lose* oral reading fluency. A theoretical possibility which might lead to this scenario would be one in which teachers pay less pedagogical attention to those already performing well at baseline, so these students do even less well in absolute terms during a second round. We believe this scenario to be highly unlikely as the top performing students would need to lose oral reading fluency (as opposed to just maintain their level through classroom instruction now targeted at lower levels), and teachers would need to have active incentives to pay less attention to high performers at baseline. As described in the introduction, the opposite seems to be more prevalent: in the absence of interventions, the status quo is for teachers to target high performers at baseline.

15 Note that these patterns hold if the Gini is calculated excluding all the children with scores of 0. The Gini coefficients for Runyankore-Rukiga, Luganda, Lhukonzo and Lugwere respectively are 0.279, 0.374, 0.530, and 0.434.

16 For reference, the OECD average in 2015 was 0.20. For other non-OECD members in 2015 the values ranged widely, but were often higher than the OECD average. For example, the ICC for Colombia was 0.25, for Indonesia 0.34, and Vietnam 0.45. Some countries performing below the OECD average were Costa Rica with 0.18, Uruguay with 0.14, and Albania with 0.19.

17 Note that when these results are displayed using “share of students at 0 cwpm” as opposed to oral reading fluency measured through cwpm, the results are very similar. These results are displayed in Table A1 in the appendix.

18 de Galbert (2021)) displays an interesting model of how language transfer happens across English, Luganda, and Runyankole-Rukiga Uganda among students. The main result highlights that transfer of basic “building blocks” of literacy across these languages does happen. Additionally, Nakamura et al. (2019) in India find the effect of literacy interventions in English may depend on whether the students have already passed a certain literacy threshold in their mother language. This finding can have implications for how we think about students performing differentially across languages (as the correlation between performance across languages might be higher than expected), ultimately shaping the different levels of learning inequality, depending on the actual language being tested.

19 These results come from regressing oral reading fluency (as measured in correct words per minute in the respective colonial language) on socioeconomic status (as measured by the variable derived from the principal component analysis). The specific sub-groups used were grades 2 in Kenya, and 4 in Uganda, although as expected, similar results hold for other sub-populations.

20 So as not to clutter the paper more, we also performed robustness checks through other graphical analysis to further confirm that the learning landscape improved in terms of percent scoring zero, overall level, and overall inequality. These analyses can be provided upon request.

21 It remains to be seen whether programs aimed at the most vulnerable participants are even more effective at reducing inequality.
